# Supplementary material for: WordCommentsAnalyzer: A windows software tool for qualitative research
Source: F1000Res. 2018 Sep 4;7:536. Originally published 2018 May 3. [Version 2] doi: 10.12688/f1000research.14819.2 (PMC6137411; doi:10.12688/f1000research.14819.2)
Supplement: Supplementary file 3 [file f1000research-7-17614-s0002.tgz › b2a79496-4ada-47c4-9682-a99ce867836d.zip!/Eight Iranian Health Journals/Archives of Rehabilitation_13.docx]

© Archives of Rehabilitation (RJ). University of Social Welfare and Rehabilitation Sciences, Koodakyar Alley, Daneshjoo Blv., Evin, Tehran, Iran. This work is licensed under [CC BY-NC 4.0](https://creativecommons.org/licenses/by-nc/4.0/).

Volume & Issue: دوره 16 - شماره 1

Title: بررسی اثربخشی گفتار نشانه‌دار بر مهارتهای زبانی حفظ موضوع ، اطلاعات اصلی و توالی وقایع داستان در دانش‌آموزان کم شنوای پیش زبانی با عمل کاشت حلزون دیرهنگام

http://rehabilitationj.uswr.ac.ir/article-1-1511-fa.html

هدف: کاشت حلزون تأثیر بسیارمثبتی بر روی رشد زبان بیانی در کودکان کم‌شنوای شدید دارد. کارایی گفتار نشانه‌دار نیز در بسیاری پژوهش‌ها موردمطالعه قرار گرفته است. هدف این مطالعه بررسی اثربخشی گفتار نشانه‌دار بر روی حفظ موضوع، اطلاعات اصلی و توالی وقایع داستان در دانش‌آموزان کم‌شنوای پیش‌زبانی با عمل کاشت حلزون دیرهنگام بود.  روش‌ بررسی: پژوهش حاضر از نوع آزمایشی بود و طرح پیش‌آزمون-پس‌آزمون استفاده شد. نمونه پژوهش به‌صورت نمونه‌گیری دردسترس انتخاب شدند. آزمودنی‌ها 9 دانش‌آموز کم‌شنوای پیش‌زبانی کاشت حلزون‌شده دیرهنگام بین سنین 11 تا 16 سال و نه ماه بودند که از مرکز کاشت حلزون لقمان انتخاب شدند. ابزار پژوهش آزمون بازگویی داستان بود. برنامه آموزش گفتار نشانه‌دار در قالب 16 جلسه 60 دقیقه‌ای به‌مدت 8 هفته به اجرا درآمد و درپایان، دوباره آزمودنی‌ها موردارزیابی قرار گرفتند. در این مطالعه از آزمون Z کولموگروف-اسمیرنوف و از آزمون تی وابسته برای مقایسه گروه‌ها استفاده شد. یافته‌ها: نتایج بررسی‌ها نشان داد آموزش گفتار نشانه‌دار بر حفظ موضوع، اطلاعات اصلی و توالی وقایع داستان در دانش‌آموزان کم‌شنوای پیش‌زبانی با عمل کاشت حلزون دیرهنگام دانش‌آموزان تأثیر مثبت و معناداری دارد (P<0/01). نتیجه‌گیری: استفاده از گفتار نشانه‌دار در برنامه توان‌بخشی کودکان کم‌شنوای پیش‌زبانی با کاشت حلزون دیرهنگام می‌تواند برای مهارت‌های زبانی حفظ موضوع، اطلاعات اصلی و توالی وقایع داستانی آنان مفید باشد.

Title: ارتباط بین رفتار شهروندی-سازمانی با تعهد سازمانی و ابعاد آن در کارکنان ستاد سازمان بهزیستی شهر تهران 1392

http://rehabilitationj.uswr.ac.ir/article-1-1516-fa.html

هدف: هدف از پژوهش حاضر، تعیین ارتباط رفتار شهروندی-سازمانی با تعهد سازمانی و ابعاد آن در کارکنان ستاد سازمان بهزیستی شهر تهران در سال 1392 است. روش بررسی: در این مطالعه توصیفی-مقطعی، 108 نفر از کارکنان ستادی سازمان بهزیستی شهر تهران به‌صورت نمونه‌گیری تصادفی ساده و طبق فرمول حجم نمونه و مطالعات گذشته، انتخاب گردیدند. داده­ها با استفاده از دو پرسشنامه استاندارد رفتار شهروندی-سازمانی و پرسشنامه تعهد سازمانی که شامل سه بُعد تعهد عاطفی، تعهد هنجاری و تعهد مستمر است، جمع­ آوری و با استفاده از آمار توصیفی، محاسبه ضریب همبستگی پیرسون و آزمون تحلیل واریانس موردتجزیه قرار گرفت. یافته‌ها: نتایج همبستگی نشان­ داد که بین رفتار شهروندی-سازمانی و تعهد سازمانی کارکنان، رابطه مثبت و معناداری (r=0/325 , P0/01&ge) وجود دارد. همچنین بین ابعاد تعهد سازمانی، تعهد عاطفی و تعهد هنجاری با رفتار شهروندی-سازمانی دارای ارتباط معنادار و مثبت بوده ­است، اما رفتار شهروندی-سازمانی با تعهد مستمر، ارتباط معناداری نداشت. نتایج تحلیل واریانس نشان ­داد که میانگین رفتار شهروندی-سازمانی برحسب سن، نوع استخدام و سابقه‌کار کارکنان، میانگین تعهد مستمر برحسب نوع استخدام و میانگین تعهد عاطفی با سابقه‌کار کارکنان دارای اختلاف معنادار بود. نتیجه‌گیری: براساس یافته­ های پژوهش نتیجه می­گیریم با ارتقای مفهوم رفتار شهروندی-سازمانی می‌توان میزان تعهد سازمانی کارکنان ستاد سازمان بهزیستی شهر تهران را افزایش داد.

Title: آیا تمرینات یکپارچگی حسّی-حرکتی بر تعادل ایستا و پویای کودکان کم‌توان ذهنی تربیت‌پذیر تأثیر دارد؟

http://rehabilitationj.uswr.ac.ir/article-1-1316-fa.html

هدف: هدف از پژوهش حاضر بررسی تأثیر تمرینات یکپارچگی حسّی-حرکتی بر تعادل ایستا و پویای کودکان کم‌توان ذهنی تربیت‌پذیر بود. روش‌ بررسی: در این مطالعه نیمه‌تجربی، به روش نمونه‌گیری هدفمند 40 نمونه از مرکز توان‌بخشی و آموزشی گل‌های بهشت شهر ارومیه با میانگین سنی 2/81±8/48 سال و ضریب هوشی 6/81±30/62 موردمطالعه قرار گرفتند. از همه کودکان پیش‌آزمون گرفته شد. سپس نمونه‌ها براساس دو فاکتور ضریب هوشی و نمرات پیش‌آزمون به دو گروه هم‌تراز (همگن) تقسیم و به‌طورتصادفی تحت‌عنوان گروه‌های آزمایش و کنترل در نظر گرفته شدند. هر گروه شامل 10 دختر و 10 پسر بود. برای ارزیابی بخش‌های مربوط به تعادل از آزمون تبحّر حرکتی برینینکس ازرتسکی استفاده شد. گروه آزمایش در طی 50 جلسه، هر جلسه 45 دقیقه و 6 بار در هفته تحریکات حسّی را تجربه و تمرینات بدنی را اجرا کردند. گروه کنترل از برنامه‌های آموزشی کلاس استفاده نمودند. یافته‌ها: پس از 50 جلسه از هر دو گروه آزمایش و کنترل، پس‌آزمون به‌عمل آمد و داده‌ها به‌وسیله آزمون مان‌ یو‌ ویتنی تجزیه و تحلیل و تفاوت معناداری در نمرات پس آزمون بین گروه‌های آزمایش و کنترل مشاهده شد (P<0/05). نتیجه‌گیری: این پژوهش نشان داد که تمرینات یکپارچگی حسی-حرکتی تعادل پویا و ایستا را بهبود می‌بخشد.

Title: معلولیت (ناتوانی) در ایران: شیوع، ویژگی‏ها و همبسته‏‌های اقتصادی و اجتماعی آن

http://rehabilitationj.uswr.ac.ir/article-1-1454-fa.html

هدف: این مقاله به‌دنبال مطالعه‏ شیوع معلولیت در ایران، ویژگی‏ها و همبسته‏های اقتصادی و اجتماعی آن با استفاده از نتایج سرشماری 1385 است. روش بررسی: در این مطالعه، از روش تحلیل ثانویه داده‏های خُرد سرشماری 1385 استفاده شد. این داده‏ها در دو سطح فردی و خانوار در قالب نمونه دو درصدی از کل کشور، جمع‌آوری شده است. در این داده‏ها، تعداد 19848 نفر دچار معلولیت و 1235445 نفر فاقدمعلولیت و همچنین در سطح خانوار، 17166 خانوار دارای حداقل یک فرد معلول و 298741 خانوار بدون فرد معلول بوده‏اند. برای تجزیه و تحلیل داده‏ها از آزمون‌های آماری دو و چندمتغیری از جمله رگرسیون لجستیک در محیط نرم‌افزار SPSS استفاده شد. یافته‏‌ها: شیوع معلولیت در ایران 14/4 در هزار برآورد شد و میزان شیوع معلولیت در مناطق روستایی بیشتر از مناطق شهری و در میان مردان بیشتر از زنان بود. همچنین نتایج نشان داد افراد دچار معلولیت، وضعیت اقتصادی و اجتماعی پایین‏تر و نامناسب‏تری در مقایسه با افراد فاقدمعلولیت داشتند. به‌علاوه، میزان و شیوع معلولیت برحسب ویژگی ‌ های سرپرست خانوار، وضعیت اقتصادی و رفاه خانوار به‌طورمعناداری متفاوت بود. نتایج آزمون رگرسیون لجستیک نشان داد که زن سرپرست بودن، تحصیلات و موقعیت شغلی پایین سرپرست خانوار، پایین‌بودن سطح رفاه خانوار و سکونت روستایی خانوار احتمال داشتن فرد معلول در خانوار را به‌طورمعناداری افزایش داد. نتیجه‏‌گیری: وضعیت رفاهی سطح پایین زندگی و ابعاد مختلف فقر در خانوار با احتمال وجود فرد دچار معلولیت در ارتباط است؛ بنابراین، سیاستگذاری‏های رفاهی برای خانوارهای کم‌درآمد و آسیب‌پذیر جامعه و ارائه بسته‏های خدمات‏رسانی، به‌ویژه نیازهای افراد دارای معلولیت، در راستای کمک به آنان و جامعه پیشنهاد می‏شود .

Title: بررسی حس وضعیت گردن در افراد مبتلا به جلوآمدگی سر و مقایسه آن با افراد سالم

http://rehabilitationj.uswr.ac.ir/article-1-1539-fa.html

هدف: یکی از گسترده‌ترین حالت‌های معیوب ستون‌ فقرات گردنی جلوآمدگی سر (FHP) است. با توجه به تغییرات بیومکانیکی عضلات و لیگامان‌های ناحیه گردن که منبع غنی مکانورسپتورهاست، در جلوآمدگی سر این امکان وجود دارد که در افراد مبتلا به جلوآمدگی سر حس وضعیت دچار اختلال شود. بررسی بازسازی زوای ا در ناحیه گردن می‌تواند به‌عنوان شاخصی برای ارزیابی حس وضعیت این ناحیه به‌کار رود . هدف از این مطالعه بررسی حس وضعیت گردن در افراد مبتلا به جلوآمدگی سر در صفحه ساجیتال و مقایسه آن با افراد سالم بود. روش بررسی: مطالعه حاضر از نوع مورد-شاهدی بود و افراد از طریق نمونه‌گیری ساده از بین دانشجویان دانشگاه علوم پزشکی ایران انتخاب شدند. 18 فرد مبتلا به جلوآمدگی سر (میانگین سن 18/23 سال) و 22 فرد سالم (میانگین 72/22 سال) در این مطالعه شرکت داشتند. برای مشخص‌شدن میزان جلوآمدگی سر عکس‌برداری از نمای ساجیتال در حالت ایستاده انجام و زاویه کرانیوورتبرال محاسبه گردید و زوایای کمتر از 49 درجه به‌عنوان جلوآمدگی سر در نظر گرفته شد. خطای بازسازی زوایه هدف (%50 دامنه حرکتی) و زاویه بی‌طرف در صفحه ساجیتال با استفاده از دستگاه تحلیل حرکت و با چشم بسته در حالت نشسته اندازه‌گیری شد. خطای مطلق و ثابت در بازسازی محاسبه و مورد تجزیه و تحلیل قرار گرفت. یافته‌ها: خطای مطلق در بازسازی‌ها تفاوت معناداری را نشان نداد (P>0/05)، اما خطای ثابت در بازسازی وضعیت بی‌طرف در بازگشت از خم‌شدن سر به سمت جلو در بین دو گروه تفاوت معناداری داشت (P<0/05). همچنین ارتباط معناداری بین شاخص توده بدنی و زاویه کرانیوورتبرال دیده شد. بدین صورت که هرچه شاخص توده بدنی بیشتر باشد زاویه کرانیوورتبرال کوچکتر است (P<0/05). نتیجه‌گیری: نتایج مطالعه حاضر نشان داد که افراد دچار جلوآمدگی سر، در بازسازی برخی حرکات ناحیه گردن خطای بیشتری نسبت به افراد سالم دارند.

Title: تأثیر سطوح بافت‌دار در فضای داخل کفش بر حس عمقی مفصل مچ پا و تعادل عملکردی در افراد دچار افتادن

http://rehabilitationj.uswr.ac.ir/article-1-1632-fa.html

هدف: یکی از علل کاهش تعادل، افت احساس در کف پا گزارش گردیده است؛ بنابراین، از کفی‌های دارای سطوح بافت‌دار برای افزایش حس کف پا و بهبود تعادل استفاده می‌شود. بیشتر مطالعات قبلی، بر روی کف پا متمرکز بوده و این تأثیرات روی نواحی دورسوم پا موردتوجه قرار نگرفته است. هدف از این مطالعه، مقایسه اثر سطوح بافت‌دار در قسمت‌های مختلف داخل کفش بر تعادل و حس عمقی مجموعه پاست. روش‌ بررسی: 16 زن و 14 مرد دارای سابقه زمین‌خوردن در یک‌سال گذشته، به‌صورت تصادفی تحت چهار حالت مداخله حسی (بافت در کف پا، بافت در زبانه و یقه کفش، کفی پلاسبو و حالت کنترل) قرار گرفتند و حس درک موقعیت مفصل مچ پا و آزمون‌های تعادل عملکردی شامل: دسترسی به قدام، برخاستن و رفتن زمان‌دار در آنها اندازه‌گیری شد. برای مقایسه داده‌های ثبت‌شده در حالات مختلف مداخله از آزمون آماری تحلیل واریانس با اندازه‌های مکرر استفاده شد. یافته‌ها: خطای حس موقعیت مفصل مچ پا در مداخله به‌شکل کفی بافت‌دار در زیر پا کاهش یافت (P<0/01). همچنین زمان انجام آزمایش برخاستن و رفتن هنگام استفاده از سطح بافت‌دار روی دورسوم، نشان از کاهش داشت (P<0/01). نتیجه­‌گیری: نتایج این مطالعه بر کاربرد سطوح بافت‌دار در داخل کفش و افزایش محتوای حس پیکری و بهبود وضعیت تعادل عملکردی و حس درک موقعیت مفصل پا تأکید دارد.

Title: بررسی مقایسه‌ای مهارت‌های حرکتی درشت، ظریف و تعادل بدنی در کودکان کم‌توان ذهنی، اوتیسم و اختلال یادگیری با کودکان عادی

http://rehabilitationj.uswr.ac.ir/article-1-1521-fa.html

هدف: هدف این پژوهش مقایسه مهارت‌های حرکتی و تعادل بدنی در کودکان کم‌توان ذهنی، اوتیسم و اختلال یادگیری با کودکان عادی بود . روش بررسی: این مطالعه از نوع توصیفی-مقایسه­ای (مقطعی) بود که بر روی 120 کودک، شامل: 30 کودک عادی، 30 کودک دارای اختلال یادگیری، 30 کودک اوتیسم و 30 کودک کم‌توان ذهنی با میانگین سن 7/7 سال که به‌صورت نمونه­گیری خوشه­ای چندمرحله­ای، از میان دانش‌آموزان مدارس و مراکز ویژه کودکان با نیازهای خاص مشهد انتخاب شدند، به انجام رسید. برای سنجش مهارت­های حرکتی این کودکان از سه مقیاس سنجش مهارت­های حرکتی درشت، ظریف و سنجش مهارت تعادل بدنی استفاده شد. برای تحلیل نتایج از شاخص­های آمار توصیفی و استنباطی مانند: میانگین، انحراف‌معیار و تحلیل واریانس چندمتغیری استفاده شد. یافته‌ها: تفاوت معناداری بین مهارت­های حرکتی ظریف، درشت و مهارت تعادل بدنی، بین کودکان عادی و کودکان کم‌توان ذهنی، اوتیسم و اختلال یادگیری وجود دارد (P<0/0001). گروه کودکان با اختلال یادگیری در کل مهارت­ها بیشترین امتیاز و گروه کودکان اوتیسم کمترین امتیاز را به‌دست آوردند. نتیجه‌گیری: نتایج این مطالعه نشان داد که مهارت­های حرکتی و تعادل در کودکان دارای اختلال یادگیری، اوتیسم و کم‌توانی ذهنی، ضعیف‌تر از کودکان عادی است که این امر نشان‌دهنده لزوم افزایش آموزش­ها در کودکان با نیازهای خاص در سنین پایین‌تر است.

Title: بررسی اثربخشی بازی‌درمانی گروهی بر مهارت‌های اجتماعی کودکان پیش‌دبستانی آسیب‌دیده شنوایی

http://rehabilitationj.uswr.ac.ir/article-1-1568-fa.html

هدف: پژوهش حاضر به‌منظور بررسی اثربخشی بازی‌درمانی گروهی بر مهارت‌های اجتماعی کودکان پیش‌دبستانی آسیب‌دیده شنوایی انجام شد. روش‌ بررسی‌: این پژوهش، یک مطالعه شبه‌آزمایشی با طرح پیش‌آزمون-پس‌آزمون و گروه شاهد بود. در این پژوهش 30 پسر آسیب‌دیده شنوایی که به روش نمونه‌گیری دردسترس از مراکز پیش‌دبستانی شهرستان ورامین و قرچک انتخاب شده بودند، شرکت داشتند. آزمودنی‌ها به‌صورت تصادفی به دو گروه 15 نفری آزمایش و شاهد تقسیم شدند. گروه آزمایش، بازی‌درمانی گروهی را در 12 جلسه دریافت کردند، درحالی‌که به گروه شاهد این آموزش ارائه نشد. ابزارهای استفاده‌شده در این پژوهش آزمون ماتریس‌های پیشرونده‌ ریون رنگی و مقیاس درجه‌بندی مهارت‌های اجتماعی بود. اطلاعات جمع‌آوری‌شده با استفاده از تحلیل کوواریانس چندمتغیری تحلیل شد. یافته‌ها: نتایج تحلیل کوواریانس چندمتغیری نشان داد که بازی‌درمانی گروهی اثر معناداری بر مهارت‌های اجتماعی کودکان آسیب‌دیده شنوایی داشت (P<0/001). همچنین نتایج بیانگر اثر مثبت و معنادار بازی‌درمانی گروهی بر همه خرده‌مقیاس‌های مهارت اجتماعی (مشارکت، ابراز وجود و مهار خود) در این کودکان بود (P<0/001). نتیجه‌گیری: بازی‌درمانی گروهی می‌تواند مهارت‌های اجتماعی کودکان آسیب‌دیده شنوایی را بهبود بخشد؛ بنابراین، برنامه‌ریزی برای بازی‌‌درمانی به کودکان آسیب‌دیده شنوایی اهمیت ویژه‌ای دارد.

Title: بررسی ارتباط بین نتایج آزمون ویدئونیستاگموگرافی (VNG) با تعداد دفعات زمین خوردن سالمندان

http://rehabilitationj.uswr.ac.ir/article-1-1614-fa.html

هدف: زمین‌خوردن از جمله مهم‌ترین مشکلات دوران سالمندی است و به‌عنوان یکی از مشکلات دوران سالمندی تلقی می‌شود. زمین‌خوردن‌های پی‌درپی از علل عمده مرگ‌و‌میر در این گروه گزارش شده است و دال بر عملکرد ضعیف جسمی و شناختی است. هدف از این مقاله، بررسی ارتباط بین نتایج آزمون ویدئونیستاگموگرافی با تعداد دفعات زمین‌خوردن در سالمندان است. روش بررسی: در این مطالعه 60 سالمند بالای 65 سال شرکت کردند که 30 نفر از آنها حداقل یک‌بار سابقه زمین‌خوردن (گروه مورد) را داشت و 30 نفر دیگر سابقه زمین‌خوردن نداشتند (‌گروه شاهد‌). آزمون‌های ساکاد، تعقیب آرام، نگاه خیره، نیستاگموس وضعیتی، نیستاگموس خودبه‌خودی و کالریک که زیرمجموعه‌های آزمون VNG است، در دانشکده توان‌بخشی دانشگاه علوم پزشکی تهران انجام شد. پس از انجام آزمون، از نسخه 19 نرم‌افزار SPSS برای تجزیه و تحلیل داده‌ها استفاده گردید. یافته‌ها: براساس نتایج به‌دست‌آمده، %74 از افراد گروه مورد در آزمون کالریک عملکرد غیرطبیعی داشتند. نتایج آزمون‌های ساکاد، نگاه خیره و تعقیب آرام نیز در %60 از افراد این گروه غیرطبیعی بود (P<0/05). همچنین عملکرد افراد مبتلا به اختلالات وستیبولار مرکزی نسبت به افراد مبتلا به اختلالات وستیبولار محیطی ضعیف‌تر بود. نتیجه‌گیری: گروه شاهد در تمام زیرمجموعه‌های آزمون VNG عملکرد بهتری نسبت به گروه مورد داشتند که دال بر وضعیت بهتر سیستم وستیبولار در این گروه است.

Title: تأثیر ارتز تغییریافته Floor Reaction AFO بر توانایی راهرفتن در کودکان فلج مغزی: مطالعۀ موردی

http://rehabilitationj.uswr.ac.ir/article-1-1650-fa.html

هدف: مطالعۀ حاضر به ‌ منظور بررسی اثر ارتز تغییریافته (Floor Reaction AFO (FLRAFO بر عملکرد راه ‌ رفتن در کودک مبتلا به فلج مغزی طراحی شده است . روش بررسی: در این مطالع ه یک کودک مبتلا به فلج مغزی دوطرفه به ‌ مدت شش هفته ارتز تغییریافته را پوشید. اثربخشی فوری ارتز شش هفته بعد از استفاده از ارتز مورد بررسی قرار گرفت. پارامترهای اندازه ‌ گیری ‌ شده شامل سرعت، کادانس و طول گام، به ‌ علاوه دامنۀ حرکتی مفاصل ران، زانو و مچ پا توسط دستگاه حرکتی وایکن ثبت شد. یافته‌ها: طول گام، کادانس و سرعت راه ‌ رفتن با پوشیدن ارتز افزایش یافته است. با پوشیدن این ارتز دامنۀ حرکتی مچ پای کودک کاهش یافت. بعد از 6 هفته، میانگین دامنۀ حرکتی زانو به هنگام راه ‌ رفتن با ارتز که در ابتدا 72/13±8/36 بود به 10/1±0/43 درجه افزایش یافت. بعد از 6 هفته استفاده از ارتز به نسبت روز اول پوشیدن ارتز، زاویۀ خم ‌ شدن مفصل ران در تماس اولیۀ پاشنه و نیز میزان انتقال بازشدن در مرحلۀ ایستایی کاهش یافت. نتیجه‌گیری: ارتز تغییر ‌ یافته می ‌ تواند سبب بهبود پارامترهای راه ‌ رفتن در کودکان مبتلا به فلج مغزی شود.
